# Prevalence of Liver Cystic Echinococcosis in Türkiye: A Systematic Review of Population-Based Ultrasound Imaging Surveys

**DOI:** 10.3390/pathogens15050496

**Published:** 2026-05-04

**Authors:** Cuneyt Kayaalp, Basak Kayaalp, Cemalettin Aydin, Servet Karagul

**Affiliations:** 1 Department of General Surgery, Faculty of Medicine, Istanbul Atlas University, Istanbul 34408, Türkiye; 2 Faculty of Medicine, Bahçeşehir University, Istanbul 34734, Türkiye; 3 Department of General Surgery, Faculty of Medicine, Istanbul Medipol University, Istanbul 34810, Türkiye

**Keywords:** hepatic, hydatid, Türkiye, epidemiology, ultrasound, screening

## Abstract

**Background**: Cystic echinococcosis (CE) is a chronic zoonotic parasitic disease with a significant impact on public health in endemic regions. The liver is the most frequently affected organ, and ultrasound-based surveys are considered a reliable tool for detecting asymptomatic infections. As population-based data specifically addressing hepatic CE prevalence in Türkiye remain limited, we aimed to assess the prevalence of liver cystic echinococcosis in Türkiye using only ultrasound-based surveys. **Methods**: A systematic review was conducted, in accordance with the PRISMA guidelines, to estimate the prevalence of liver CE in Türkiye based exclusively on ultrasound-based field surveys. Electronic databases (PubMed and Scopus) were searched up to March 2026. Eligible studies included population-based human screening surveys reporting hepatic CE prevalence confirmed via ultrasonography. Data were extracted and descriptively pooled, with subgroup analyses performed according to age group (children vs. adults) and residential setting (urban vs. rural). The protocol was prospectively registered in the International Platform of Registered Systematic Review and Meta-analysis Protocols (INPLASY; registration Number: INPLASY202630029). Only human ultrasound-based screening studies including liver CE data were accepted; alveolar echinococcosis studies were excluded. Pooled prevalence estimates with 95% confidence intervals (CIs) were calculated using a random-effects model, and between-study heterogeneity was assessed with the I^2^ statistic. **Results**: We analyzed the data of 23,154 people from 11 different provinces reported in 8 studies. The overall pooled liver CE prevalence was 0.31% (95% CI: 0.14–0.54), while it was 0.12% and 0.43% for urban and rural residents, respectively. Adults had higher prevalence of liver CE than children (0.43% vs. 0.16%). When separated by both living area and age, the prevalence rates were as follows: urban children 0.07%, urban adults 0.21%, rural children 0.29% and rural adults 0.60%. **Conclusions**: This is the first systematic review evaluating the prevalence of liver CE in Türkiye exclusively from ultrasound-based studies. While the overall prevalence of liver CE was 0.31%, adults living in rural areas presented a nearly two-fold higher rate (0.60%). Ultrasound-based screening provides a practical and effective approach for epidemiological surveillance. Targeted control strategies—including community-based screening, health education, and veterinary interventions—are essential to reduce transmission and disease burden, particularly in high-risk rural populations.

## 1. Introduction

Cystic echinococcosis (CE) is a chronic zoonotic parasitic disease with a global distribution. It is primarily transmitted between canids (definitive hosts) and livestock (intermediate hosts), with humans acting as accidental hosts. The disease has a significant public health impact in endemic regions, leading to considerable morbidity and economic losses [1].

Türkiye, located at the crossroads of Europe and Asia, has diverse geographic and climatic conditions that influence the prevalence and transmission dynamics of CE [2]. Despite various control efforts, the disease remains a persistent public health challenge. CE is most commonly located in the liver and ultrasound-based surveys have emerged as a valuable epidemiological tool for its detection, offering high sensitivity and specificity, particularly in asymptomatic individuals. Ultrasound-based CE surveys have already been carried out in different provinces, reflecting various epidemiological patterns (rural, urban or mixed) [3,4,5]. However, none of the studies conducted to date were specific to the liver, the organ most commonly affected by CE. A country-specific synthesis for Türkiye is particularly informative for three reasons: (i) Türkiye spans several endemic eco-climatic zones with markedly different animal husbandry practices, which makes pooled international estimates less useful for domestic policy; (ii) Türkiye has one of the largest bodies of ultrasound-based CE survey data in the world, enabling age- and residence-stratified estimates that are not feasible in most countries; and (iii) as public health decisions on where and how to deploy screening are made at the national and provincial level, nationally pooled, subgroup-specific estimates are needed. The findings can further be interpreted alongside the broader international literature on CE in endemic regions [2,6,7,8]. This systematic review synthesizes available ultrasound-based studies to determine the overall prevalence of liver CE in Türkiye, as well as in rural/urban regions separately.

## 2. Methods

### 2.1. Search Strategy and Data Sources

**Figure 1:**
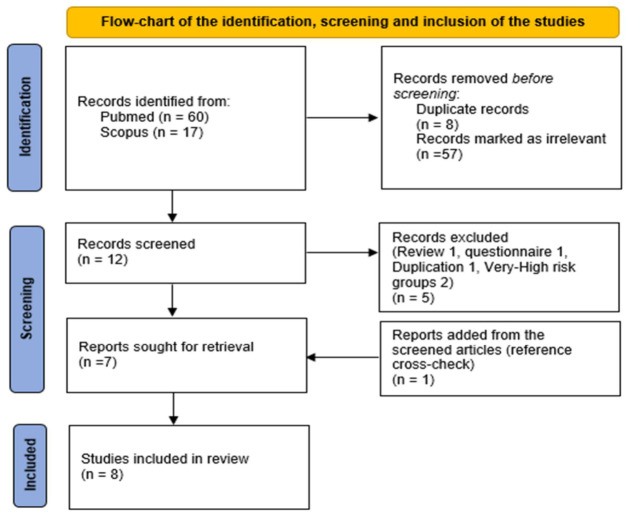
Preferred Reporting Items for Systematic Reviews and Meta-Analysis (PRISMA) flowchart.

A systematic literature review was conducted using the electronic databases PubMed and Scopus in March of 2026. There were no restrictions on article publication dates. The search was performed using the following keywords in the title, abstract or keywords of the articles: (echinococ* OR hydati*) AND (Turkey OR Türkiye OR Turkiye) AND (ultraso*) AND (prevalence). Articles were searched by two authors (C.K. and B.K.) and, following the selection of the studies, their references were cross-checked for any missing articles (Figure 1). Disagreements were settled via discussions with the third and fourth authors (C.A. and S.K.) until an agreement was reached. If there was any data missing from an article, we contacted the authors via e-mail or phone. The review followed the PRISMA 2020 guidelines, and the protocol was prospectively registered in the International Platform of Registered Systematic Review and Meta-analysis Protocols (INPLASY; registration Number: INPLASY202630029). The PICOS framework was used to structure the research questions and guide the selection of appropriate studies.

P (Participants): All population groups, regardless of age and gender, were included in the systematic review.

Studies were included if they:

- Were ultrasound-based epidemiological surveys conducted in Türkiye.
- Reported prevalence rates only in human populations.
- Provided sufficient data on sample size, methodology, and diagnostic criteria.

Studies were excluded if they:

- Were case reports, questionnaires, reviews, or experimental studies.
- Lacked ultrasound confirmation of CE.
- Focused solely on veterinary cases without human epidemiological data.
- Cases with Echinococcus multilocularis (alveolar echinococcosis).
- Prevalence studies focused on very-high-risk groups (e.g., family members of CE patients).

I (Intervention): Studies that included ultrasound scanning along with serology were included in the review, while those that only performed screening through serological studies were excluded. Single-arm screening studies with ultrasound or the relevant arm of multi-arm studies were included in the analyses.

C (Comparison): As the risk factors in individuals with and without the disease were not the subject of this study, we separated the living regions of the populations into rural and urban, as the prevalence was expected to differ between the two settings. Additionally, we analyzed the prevalence in children and adults due to the time-dependent features of CE.

O (Outcome): Only the prevalence of liver hydatid cysts was investigated. Where available, ultrasonographic findings were recorded according to the WHO-IWGE ultrasound classification (CE1–CE5), in order to distinguish active (CE1–CE3b) from inactive or past infection (CE4–CE5); however, as most included studies reported only the number of liver cysts without cyst stage data, stratified meta-analysis by cyst stage was not feasible. Although cases diagnosed with other organ locations were also added to the tables, they were not included in the statistical analysis. Population geography, research affiliation, population demographics (number, age and gender), the characteristics of the ultrasound device used in the scan, prevalence rate and the characteristics of the region were recorded.

S (Study Design): Cohort studies and community-based cross-sectional ultrasound surveys were included. Retrospective reports of hospital data were not included in this systematic review.

### 2.2. Statistical Analysis

Pooled prevalence estimates with 95% confidence intervals (CIs) were calculated using a random-effects meta-analysis based on the DerSimonian–Laird estimator. As several included studies reported very low event counts, individual study proportions were transformed using the Freeman–Tukey double-arcsine transformation prior to pooling, then back-transformed for reporting. Between-study heterogeneity was assessed with Cochran’s Q test and quantified using the I^2^ statistic, with I^2^ values of 25%, 50% and 75% interpreted as low, moderate and high heterogeneity, respectively. Subgroup analyses were performed a priori by residence (urban vs. rural) and by age (children < 18 years vs. adults ≥ 18 years), as well as by their combinations. For studies reporting the median and range only, the mean and standard deviation were estimated using the approach described by Hozo et al. [9]. Both dichotomous and continuous variables were statistically analyzed using the chi-square test or the Fisher exact test (where expected values were less than 5) and Student’s *t*-test, where appropriate. All analyses were conducted in SPSS 13.0. A two-sided *p* value < 0.05 was considered statistically significant.

## 3. Results

**Figure 2:**
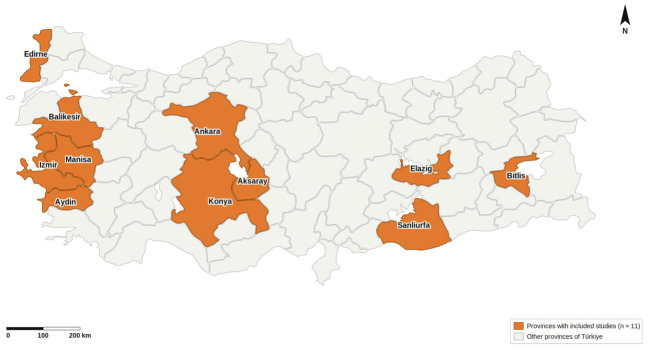
Geographic distribution of the included studies.

After the first selection, the full texts of 12 articles were examined, of which 5 were excluded (review, questionnaire alone, overlapping records with an already analyzed study or prevalence studies in very-high-risk groups; see Figure 1). One additional study was added through reference checking. Therefore, a total of 8 studies [2,3,4,5,10,11,12,13] including 23,154 people residing in 11 different provinces were enrolled (Figure 2). According to the analysis of studies with available data, 7249 men (46.5%) and 8357 (53.5%) women were screened, with age ranging between 7 and 88 years. Furthermore, according to the available data, 9863 people were living in urban areas and 13,291 in rural areas (Table 1). The studies were conducted between 1989 and 2018. Seven studies were carried out by universities in the research regions themselves, and one study was completed under the European Union project (Heracles Project). Three studies included only primary school children, four studies included only adults and one study covered both children and adults.

When we pooled the data from 8 field studies using a random-effects model, the overall liver CE prevalence was 0.31% (95% CI: 0.14–0.54), while those for urban and rural populations were 0.12% and 0.43%, respectively. Adults had a higher prevalence of liver CE than children (0.43% vs. 0.16%). The prevalence rates separated by living area and age were as follows: urban children 0.07%, urban adults 0.21%, rural children 0.29% and rural adults 0.60% (Table 2) [2,3,4,5,10,11,12,13]. Between-study heterogeneity was moderate for the pooled rural estimate and low to moderate for the other subgroups, consistent with the differences in age structure, region and sampling year.

## 4. Discussion

Our findings: Türkiye has contributed a substantial share of the published ultrasound-based field surveys and clinical case series on cystic echinococcosis, reflecting the well-documented high prevalence of the disease in the country and the long-standing research interest of Turkish groups in this field [1,2,14]. This is the first systematic review evaluating the prevalence of liver CE in Türkiye based on ultrasound studies. Notably, while the overall prevalence was 0.31%, adults living in rural areas presented a nearly two-fold higher rate of liver CE (0.60%). Nevertheless, this absolute pre-test probability remains low for unselected population-wide screening, which is an important consideration when designing programmatic interventions [2,3,4,5,10,11,12,13].

Why liver screening? First, CE is most commonly seen in the liver. Second, ultrasonography is a very reliable diagnostic method for liver cysts. A chest X-ray is required to screen for lung cysts, which requires exposing individuals to ionizing radiation (albeit in low doses). If the liver CE prevalence can be determined in a rational way, the overall number of CE cases can be estimated. For these reasons, we chose to analyze only studies examining the prevalence of liver CE using ultrasonography, a highly accurate and simple screening method [1,4,13].

Which screening method? There are several options for the screening of liver CE in the field, such as serological tests, abdominal ultrasound, hospital records and questionnaires. Serological tests have a high rate of false positives due to cross-reactivity with other parasites and require a blood sample, which may lead people to avoid testing. Hospital records rely on existing data and do not require fieldwork for the diagnosed cases; however, they do not allow for determination of prevalence in the general population. Community questionnaires help to assess risk factors and knowledge gaps, but cannot confirm infections. Ultrasound-based surveys provide an effective means for detecting liver CE compared to serological diagnosis, enabling direct visualization of cyst morphology and classification based on the WHO-IWGE classification system. The WHO-IWGE classification distinguishes active cysts (CE1, unilocular; CE2, multivesicular/multiseptated; CE3a, detached endocyst/“water-lily” sign; CE3b, solid inclusions with daughter vesicles) from transitional (CE3) and inactive stages (CE4, solid content; CE5, calcified), and separating active from inactive disease has clear therapeutic implications. Among the included surveys, only the multi-country Heracles Project explicitly reported cyst stage distributions [2], whereas most Turkish surveys have reported aggregate numbers only; therefore, we could not present a pooled stage distribution in this study. Additionally, during abdominal ultrasonography, other organs (e.g., the spleen and kidneys) can also be examined for CE. In Türkiye, hepatic hydatid disease remains a common surgical condition, with morbidity and length of stay varying with surgical technique [1,15]. Very-high-risk groups such as household contacts of operated cases can have higher detection rates and may require targeted approaches [16]. Ultrasound screening for liver CE is a safe, non-invasive, and radiation-free method; however, it requires trained personnel and equipment. For an ideal comprehensive survey of liver CE, an approach using ultrasound should be the mainstay, while serology is recommended for differential diagnosis following ultrasound imaging. This approach should be supported by hospital records for previously diagnosed patients, while risk factor analysis should be achieved through questionnaires [4,13,17].

Temporal change in prevalence: Although the available ultrasound-based surveys span almost three decades (1989–2018), they were performed in different settings and age groups, which limits any direct inference regarding time trends. Studies focusing on school children and university students (generally younger and more urban/organized populations) reported lower prevalence (roughly 0.11–0.40%), whereas surveys in adults from rural communities reported higher rates (0.53% in Konya in 1989 and 0.59% in the 2018 multi-province rural survey). Importantly, a non-zero prevalence in children (especially in rural children, 0.29%) indicates that transmission continues, while the higher prevalence in adults likely reflects the cumulative nature of CE with slow cyst growth and long asymptomatic periods. Overall, rather than a clear decrease over time, the observed variability appears to be driven mainly by differences in age structure, rural exposure and geographic sampling.

Estimation of total CE prevalence: As the liver is the most frequently involved organ in cystic echinococcosis, the prevalence of liver CE can be used as a pragmatic proxy to approximate the overall CE burden in the population. If one assumes that hepatic involvement represents about 70% of all human CE localizations, the total CE prevalence can be approximated by dividing the liver prevalence by 0.70 (i.e., multiplying by ~1.42). Using this approach, the pooled liver CE prevalence of 0.31% corresponds to an estimated overall CE prevalence of approximately 0.44%. For rural adults, a liver prevalence of 0.60% would translate to an estimated overall prevalence of ~0.85%. These figures should be interpreted cautiously, as organ distribution varies by setting and isolated lung or other organ cysts are not captured under an abdominal ultrasound-only strategy. Very-high-risk groups, such as household contacts of operated cases, can have higher detection rates and may require targeted approaches [1,16].

Other studies around the world: The epidemiology of CE differs markedly between and within countries, depending on animal husbandry practices, dog population control, slaughtering/meat inspection and hygiene infrastructure [14]. In endemic regions of the Mediterranean basin, the Balkans, Central Asia and parts of South America and western China, community-based ultrasound surveys consistently show two recurring patterns: (i) higher prevalence in rural/pastoral communities than in urban settings; and (ii) increasing prevalence with age. Our pooled results reproduce both patterns in Türkiye, with a roughly three- to four-fold higher prevalence in rural versus urban populations and the highest burden in rural adults. The large, multi-province ultrasound study performed within the scope of the Heracles Project also illustrates that CE prevalence can vary substantially between nearby endemic countries and between regions within the same country, emphasizing the value of standardized ultrasound protocols and harmonized reporting for cross-country comparisons [2].

Taken together, these findings suggest that single-center hospital series are likely to underestimate the true community burden, because many infected individuals remain asymptomatic for years and are detected only through active screening. Therefore, ultrasound-based field surveys—ideally combined with standardized cyst staging and parallel collection of exposure data—remain the most informative approach for monitoring transmission and evaluating the impacts of control programs.

Practical results of this study: Our findings highlight the endemic nature of liver CE in Türkiye and the need for targeted public health interventions. Although the prevalence was higher in rural adults (0.60%) than in other subgroups, the absolute pre-test probability remains low, such that indiscriminate mass ultrasound screening of all rural adults would yield approximately 1 case per 166 individuals scanned. This strategy thus carries limited programmatic efficiency and a high opportunity cost, particularly given constraints on ultrasound capacity and trained personnel. We therefore do not recommend unselected population-wide screening, and have revised our recommendation toward a risk-enriched screening strategy. Strategies should include, especially for adults living in rural areas with identified risk factors: (i) education and awareness campaigns on disease transmission and preventive measures; (ii) strengthening deworming programs for dogs and improving meat inspection practices; (iii) ensuring proper disposal of animal waste and increasing access to clean water; (iv) risk-enriched ultrasound screening of individuals with one or more of the following features—direct livestock contact, close occupational or domestic contact with dogs (especially sheep-guarding dogs), household clustering with an index case or residence in highly endemic micro-regions—rather than unselected population-wide screening; and (v) vaccination programs for livestock.

### 4.1. Limitations

In this study, only the prevalence of liver CE cases was investigated. In addition, insufficient sampling was performed in the eastern Anatolian provinces of Türkiye, where CE cases are known to be high. The included surveys cover a limited number of provinces and do not uniformly represent all seven geographic regions of the country, which restricts the national representativeness of our pooled estimates; regions with potentially higher burden, such as Eastern and Southeastern Anatolia, are particularly underrepresented. In addition, most included surveys did not report WHO-IWGE cyst stages, preventing stratified analyses by active versus inactive disease. Differences in age composition and in the balance of rural and urban participants across the included studies contribute to between-study heterogeneity, and may also affect the direct comparability of subgroup estimates.

### 4.2. Future Study Prospects

In the future, it would be appropriate to conduct a comprehensive study covering adults residing in rural areas in the Eastern Anatolia region, where CE is known to be more common in Türkiye. This study should be based on abdominal ultrasonography, thus providing additional information about alveolar echinococcosis, which is also more common in the defined region. Future studies should also systematically apply the WHO-IWGE classification to allow for separation of active from inactive disease, and should collect individual-level exposure data (livestock contact, dog ownership, water source) to enable formal evaluation of risk-enriched screening strategies and their cost-effectiveness.

## 5. Conclusions

Cystic echinococcosis (CE) remains a significant public health concern in Türkiye, with varying prevalence rates across different regions. Ultrasound-based surveys provide valuable epidemiological insights, enabling accurate disease prevalence estimates and early detection of asymptomatic cases.

Identifying high-risk populations associated with CE is essential for guiding public health interventions and informing policymakers. Such efforts can enhance surveillance systems and support the implementation of targeted preventive strategies to reduce the burden of human infection.

The highest disease burden was observed among adults residing in rural areas, highlighting the need for comprehensive control measures including community health education, strengthened veterinary control programs, and improvements in sanitation and hygiene practices. However, as the absolute prevalence is still low in this subgroup, we do not recommend unselected population-wide ultrasound screening. Rather, we recommend a risk-enriched screening strategy that focuses on rural adults with one or more identifiable exposure-related risk factors, in conjunction with awareness programs and veterinary/sanitary interventions.

## Figures and Tables

**Table 1:** Characteristics of included studies.

Author	Year	Affiliation	City	Sample Size	Population	Age (Years)	Male	Female	Urban	Rural	Liver CE	Prevalence (%)
Kalyoncu [3]	1989	Hacettepe University	Konya	190	Adults	39 ± 18 (20–71)	93	97	None	190	1	0.53
Kilimcioğlu [4]	2006	Celal Bayar University	Manisa	1205	Primary school students	10.5 ± 2.2 (7–17)	NA	NA	None	1205	4	0.33
Ok [10]	2007	Celal Bayar University	Manisa	6093	Primary school students	7–14	NA	NA	4240	1853	7	0.11
Arda [11]	2009	Ege University	İzmir	250	University students	21 ± 2 (17–29)	NA	NA	250	None	1	0.40
Bakal [5]	2012	Fırat University	Elazığ	2314	Primary school students	10.9 ± 2.2 (7–14)	1217	1097	1098	1216	3	0.13
Ertabaklar [12]	2012	Adnan Menderes University	Aydın	209	Adults and children	46.8 ± 16.9 (7–88)	80	129	None	209	1	0.48
Kilimcioğlu [13]	2013	Celal Bayar University	Manisa	4275	University students	20.7 ± 1.5	2040	2235	4275	None	7	0.16
Tamarozzi [2]	2018	Heracles Project	Edirne, Ankara, Balıkesir, Aksaray, Bitlis, Şanlıurfa	8618	Adults	NA	3819	4799	None	8618	51	0.59
Total				23,154					9863	13,291	75	0.32

**Table 2:** Pooled prevalence of liver CE in Türkiye by subgroup.

Prevalence of Liver CE	Sample Size	Proportion (%)	95% CI (%)
Total	23,154	0.310	0.145–0.536
Children	9612	0.164	0.078–0.283
Adults	13,333	0.425	0.144–0.852
Urban	9859	0.126	0.048–0.214
Rural	13,286	0.428	0.272–0.619
Urban children	5334	0.071	0.017–0.161
Urban adults	4525	0.206	0.062–0.435
Rural children	4269	0.288	0.150–0.471
Rural adults	8808	0.601	0.450–0.773

## Data Availability

All data generated or analyzed during this study are included in this published article and its Supplementary Information files.

## Supplementary Materials

The following supporting information can be downloaded at: https://www.mdpi.com/article/10.3390/pathogens15050496/s1, File S1: PRISMA checklist [18].

## Abbreviations

The following abbreviations are used in this manuscript:

CE	Cystic echinococcosis
CI	Confidence interval
INPLASY	International Platform of Registered Systematic Review and Meta-analysis Protocols
NA	Not available
PICOS	Population, Intervention, Comparison, Outcome, Study Design
PRISMA	Preferred Reporting Items for Systematic Reviews and Meta-Analyses
SPSS	Statistical Package for the Social Sciences
WHO-IWGE	World Health Organization Informal Working Group on Echinococcosis
